# Assessing Groundwater Potential in the Ziway Lake Watershed Using Geographical Information System, Analytic Hierarchy Process, and Drinking Water Quality Index

**DOI:** 10.1002/gch2.202400354

**Published:** 2025-04-27

**Authors:** Tariku Takele, Abraham Mechal, Berihu Abadi Berhe

**Affiliations:** ^1^ Department of Geology College of Natural and Computational Sciences Dilla University P.O. Box 419 Dilla Ethiopia; ^2^ Geology Department Addis Ababa Science and Technology University (AASTU) P.O. Box 16417 Addis Ababa Ethiopia; ^3^ Mineral Exploration, Extraction, and Processing Center of Excellence (MEEP) AASTU P.O. Box 16417 Addis Ababa Ethiopia; ^4^ School of Earth Sciences College of Natural and Computational Sciences Mekelle University P.O. Box 231 Mekelle Ethiopia

**Keywords:** geospatial, groundwater potential, lake Ziway, MCDA, SWAT

## Abstract

This study investigates the groundwater potential zones in the Ziway Lake watershed of Ethiopia's rift valley using a GIS‐based multi‐criteria decision analysis within the Analytical Hierarchy Process (AHP). Environmental factors, including recharge, lithology, elevation, lineament density, and drainage density, are analyzed. The soil and water assessment tool (SWAT) model is applied to estimate groundwater recharge for the watershed. The performance of the SWAT model, evaluated based on observed streamflow at Katar and Meki stations, demonstrates good performance during the calibration and validation phases. The watershed's groundwater potential is classified as Very low (18.01%), Low (17.62%), Moderate (12.05%), High (28.34%), and Very High (23.98%). Groundwater potential zones are integrated with drinking water quality index zones using GIS, showing that (39.72%) of very high and high potential areas have good to excellent water quality. Results show that over half of the watershed has moderate to very high groundwater potential, identifying critical areas for sustainable water management. The findings provide useful guidance for identifying key areas for groundwater exploration and conservation, offering a practical approach that can be applied to other regions to ensure sustainable management.

## Graphical abstract

This study identifies groundwater potential zones in Ethiopia’s Ziway Lake watershed using GIS and the SWAT model. Over half the area shows moderate to very high potential. Nearly 40% of high‐potential zones also have good to excellent water quality. The results offer a practical tool for sustainable groundwater management, with potential application to another region.

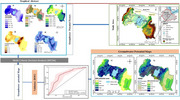

## 1 Introduction

Groundwater is free of contamination, able to buffer climate fluctuation, cheaper to develop, and adaptable in demand‐based application under field circumstances relative to surface water sources.^[^ ^1^, ^2^, ^3^ ^]^ As a result, it is a highly preferred resource for domestic, agricultural, and industrial purposes.^[^ ^4^ ^]^ However, the distribution, availability, and accessibility of this groundwater are erratic, both in space and time.^[^ ^5^ ^]^ Groundwater potentiality is crucial for maintaining aquifer levels and ensuring water supplies, particularly in arid regions with limited surface water. Several surface and subsurface attributes can influence the distribution and occurrence of groundwater regimes at various scales.^[^ ^6^, ^7^, ^8^ ^]^ These factors include soil, land cover, topography, climate, and agriculture.^[^ ^9^, ^10^, ^11^, ^12^ ^]^ The underlying lithology characteristics, lineaments, landforms, soil texture, land use, land cover types, and rainfall also play a significant role.^[^ ^13^ ^]^

Mapping groundwater potential zones (GWPZ) is crucial for effective water resource management, enabling targeted artificial recharge and groundwater development to ensure sustainable replenishment and long‐term water security.^[^ ^14^, ^15^ ^]^ There are several methods for mapping groundwater potential, but Analytical Hierarchy Process (AHP) stands out as a reliable and flexible tool for analyzing key factors using available and remote sensing data^[^ ^16^, ^17^ ^]^ The combined application of geospatial technologies, including geographical information systems (GIS) and remote sensing (RS), has been increasingly utilized to improve the understanding of groundwater potential.^[^ ^12^, ^18^ ^]^ These technologies decompose complex datasets for better visualization, revealing patterns and relationships critical to assessing groundwater potential.^[^ ^19^ ^]^ The integrated application of the AHP, RS, and GIS methods enhances decision‐making by prioritizing factors according to their relative importance^[^ ^20^, ^21^ ^]^ In contrast, traditional exploration techniques such as hydrological testing, ground surveys, geophysical methods, and extensive drilling are resource‐intensive and time‐consuming.^[^ ^22^, ^23^ ^]^ Geospatial technologies have become widely adopted due to their simplicity and practicality, offering valuable preliminary insights that inform decision‐making before investing in expensive field explorations.^[^ ^24^ ^]^

The hydrogeology of Ethiopia is variable and partly related to the complex hydrogeological setting of the country caused by the variability in climate, soil, land use land cover (LULC), physiography, and lithology, as well as the disruption of lithologies by geological structures.^[^ ^25^, ^26^ ^]^ In the country, while its use for irrigation is limited, rapid population growth and agricultural expansion have increased the demand for groundwater.^[^ ^27^, ^28^ ^]^ Therefore, a clear understanding of the regional and local hydrogeological setting is required to develop and manage groundwater resources in Ethiopia. The Ziway Lake watershed (ZLW) is facing significant challenges with groundwater depletion, influenced by climate change, land use changes, and increasing water demand.^[^ ^29^, ^30^, ^31^, ^32^ ^]^ Groundwater plays a critical role in supporting both rural and urban populations, making its availability and sustainability essential.^[^ ^33^, ^34^ ^]^ However, over time, both surface water and groundwater recharge have diminished, partly due to the expansion of irrigation around the lake and adjacent rivers.^[^ ^35^ ^]^ Thus, this study aims to produce a groundwater potential map and integrate it with a drinking water quality suitability map, providing a comprehensive tool for sustainable groundwater development and management strategies using GIS‐based multi‐criteria decision analysis (MCDA). These robust and efficient groundwater potential mapping techniques are essential to reduce water scarcity and competition for water resources, ensuring equitable access to water for various uses in the region.

## 2 Study Area

**Figure 1:**
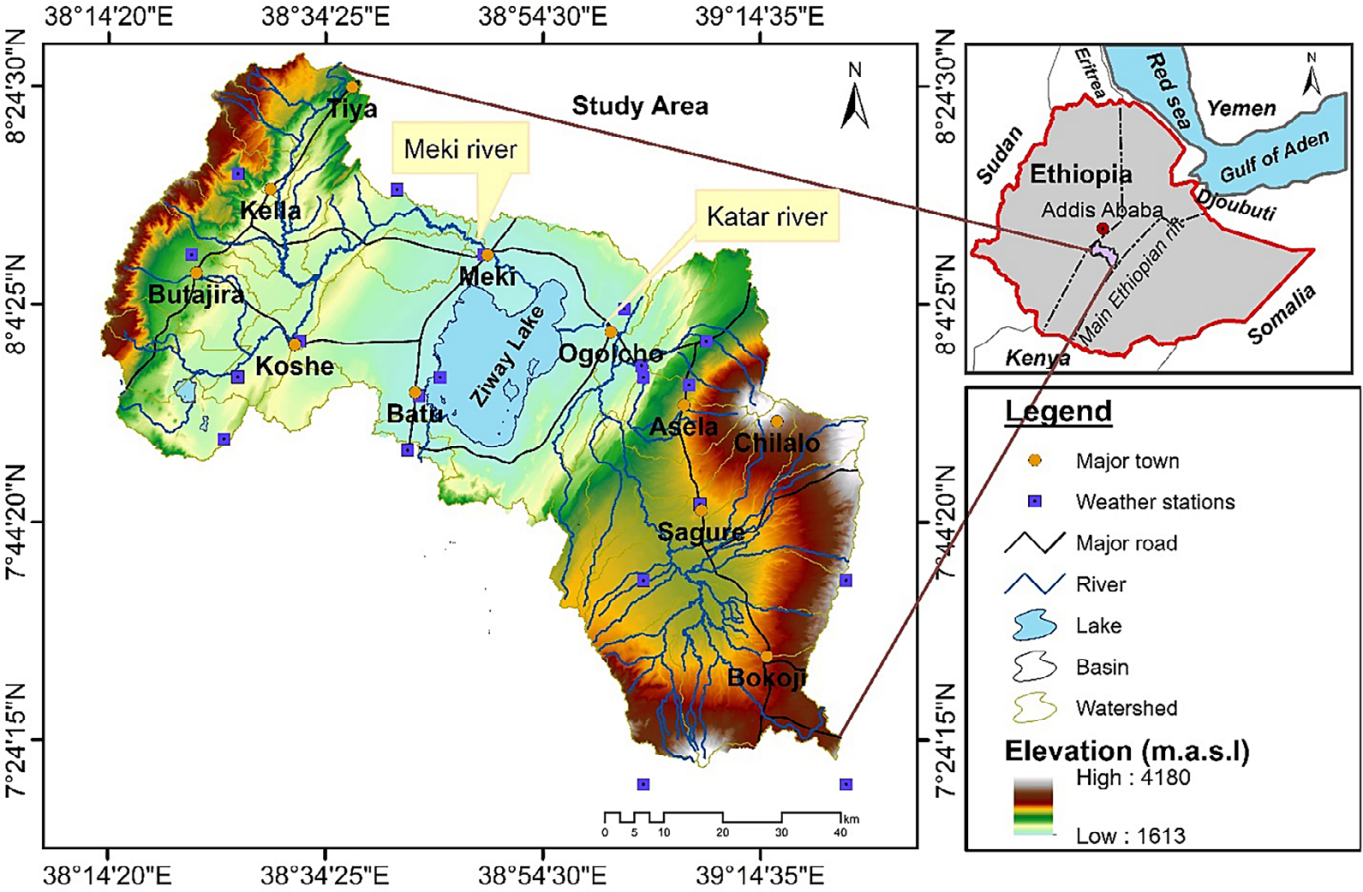
Site map of the study area showing drainage and elevation variations.

The ZLW is located within the Main Ethiopian Rift (**Figure** **1**). The area plays a crucial role in providing vital water resources to surrounding communities and ecosystems, serving as a central lifeline. The watershed covers an area of ≈7089.09 (km^2^), bounded by latitudes ranging from 7°20′54″ to 8°25′56″, and longitudes from 38°13′02″ to 39°24′01″ (Figure 1). The study area stretches from the Gurage Mountains in the west, across the Ethiopian Rift Valley, reaching areas near Mount Chilalo, Galema, and Arsi Kakka in the east. It is accessible via major asphalt roads connecting Addis Ababa with Asela, Mojo, Ziway, and Butajira, though travel is more difficult in highland and escarpment regions due to the rugged terrain.

The climate within the watershed exhibits significant variations and includes semi‐arid and arid regions, dry sub‐humid areas, and humid to dry plains. Annual rainfall ranges from ≈733 mm on the rift floor to 1100 mm in the highlands, with temperatures varying between 13.5 and 21.8 °C, depending on the location. Geographically, the area is characterized by complex landscapes, including river channels, fault formations, and mountain ranges. The eastern and western parts are dominated by volcanic mountains and step faults, while the central rift region is relatively flatter, featuring moderate slopes and volcanic hills (Figure 1). The Meki and Katar Rivers, originating from the east and west, respectively, served as the main water sources for Lake Ziway, supplemented by smaller rivers flowing from the highlands. Both surface water and groundwater, replenished by precipitation in the highlands, are crucial for the hydrology of Lake Ziway. The highland areas have a higher drainage density compared to the escarpment regions, influenced by variations in climate and slope.

The ZLW watershed features three primary lithologic groups: Precambrian and Mesozoic sedimentary rocks, volcanic and volcano‐clastic rocks, and Quaternary sedimentary deposits. These groups are further divided into seven sub‐lithologic units. Quaternary Sedimentary covers constitute Lacustrine sediments which included Sand, silt, pyroclastic deposit and Diatomite (Qt), Volcanite and Volcano‐classics (Oligocene to Middle Pleistocene), Rhyolite lava, pumice, panthelleric and obsidian flows (Qb), Basaltic lava flow and scoria cones (Qw), Rift floor Ignimbrite includes peralkaline rhyolitic ignimbrite (Pp), Chilalo Shield volcanoes constitutes Basalt, Trachyte, Mugearite and phonolite (Mt), Volcanite of plateaux, rhyolitic ignimbrite and tuff (Tt), Basement cover comprises Gneiss and sandstone, shale, marl, and limestone (Bm). These types and properties of volcanic and sedimentary rocks affect porosity and permeability, which in turn shape groundwater variability.

## 3 Datasets and Methods

### 3.1 Datasets

Groundwater potential factors were identified and weighted based on expert judgment and relevant studies.^[^ ^32^, ^36^, ^37^, ^38^, ^39^, ^40^ ^]^ Key factors included recharge estimated using the soil and water assessment tool (SWAT) model, elevation, lineament and drainage density derived from a digital elevation model (DEM), rainfall and temperature from the National Metrological Agency (NMA) and soil (from the Food and Agriculture Organization (FAO), LULC from Landsat 8 (available on the United States geological survey (USGS) webpage), geology (from the geological survey of Ethiopia (GSE)), river discharge (from the Ministry of Water and Energy of Ethiopia (MoWE)), and water points (Well and spring data from the regional Water Resource Office) (**Table** **1**).

**Table 1:** Summary of the datasets used in this study.

Datasets	Resolution	Sources and references
DEM	12.5 m × 12.5 m	Earth data (https://search.asf.alaska.edu)
Soil data	250 m × 250 m	MoWE and FAO (https://www.fao.org/home/en/)
Landsat 8	30 m × 30 m	USGS (https://earthexplorer.usgs.gov)
Geological map	250 m × 250 m	GSE (Geological Survey of Ethiopia),^[^ ^37^, ^39^ ^]^
Weather data	1982–2016	EMI (Ethiopian Meteorological Institute)
Water point data	–	Regional water resource office
River discharge	1982–2016	Ministry of Water and Energy of Ethiopia (MoWE)

**Table 2:** Sensitivity analysis, the rank, fitted value, and the ranges of parameters.

Parameter	Definitions	t‐stat	Sensitivity rank	Fitted value	Parameter range
CH_K2	Channel hydraulic conductivity (for routing	0.69	14	1.57	0–250
GWQMN	Groundwater recharge threshold	0.8	13	810.70	0–5000
SOL_AWC	Soil Available Water Content	0.82	12	−0.15	±0.25
GW_DELAY	Groundwater delay (time delay for groundwater flow)	0.89	11	8.50	0–500
GW_REVAP	Groundwater Revap (rate of recharge to the surface)	0.97	10	0.12	0.02–0.2
RCHRG_DP	Deep groundwater recharge rate	1.08	9	0.30	0–10
EPCO	Plant coefficient (evapotranspiration)	1.15	8	0.94	0–1
CANMX	Maximum canopy storage	1.2	7	4.26	0–10
REVAPMN	Minimum groundwater recharge threshold	1.75	6	244.87	0–500
ALPHA_BF	Baseflow alpha (rate of baseflow recession)	2.18	5	0.42	0­‐10
ESCO	Soil evaporation compensation factor	2.3	4	0.55	0.4–0.59
SOL_Z	Soil depth (for root zone storage)	2.65	3	0.15	±0.25
SOL_K	Soil‐saturated hydraulic conductivity	2.74	2	0	±0.25
CN2	Curve number (used for runoff calculation)	3.88	1	−0.20	±0.25

4

### 4.1 Groundwater Potential Mapping

**Figure 2:**
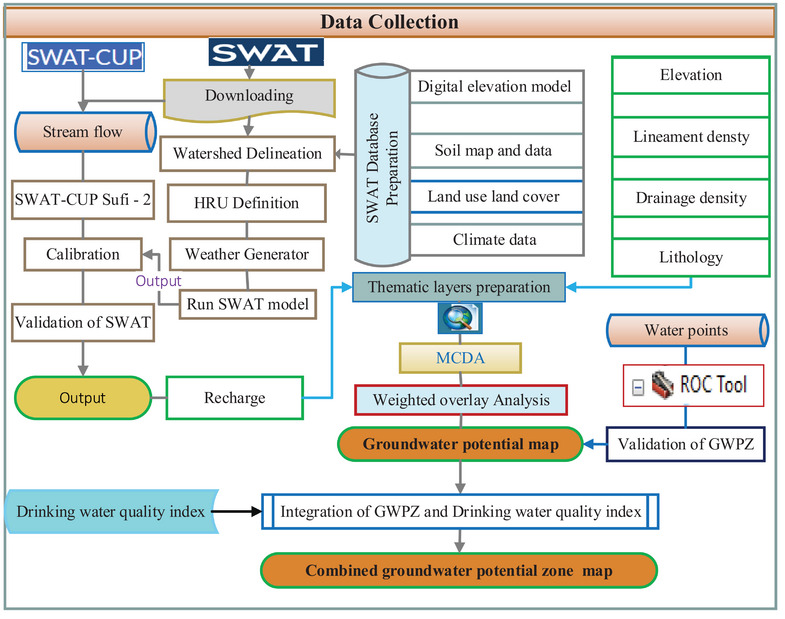
The methodological framework of the study.

This study employs a methodological framework (**Figure** **2**) combining RS, SWAT, and a GIS‐based AHP model to map groundwater potential zones. The process begins with estimating spatial groundwater recharge via the SWAT model using Climate, soil, LULC, and slope data. Thematic layers (lithology, elevation, lineament density, and drainage density) were then created and integrated with the groundwater recharge layer. These layers were reclassified, rescaled to 12.5 m, and analyzed using GIS‐based AHP techniques to delineate groundwater zones. The final groundwater potential map was validated and integrated with a drinking water quality map.

### 4.2 Soil and Water Assessment Tool (SWAT) Model

*Model Description*: The SWAT model is a free, open‐source tool designed to simulate and predict the impacts of land use on water, soil, and chemical dynamics within large watersheds. It models key hydrological processes; including rainfall, runoff, and evaporation, using daily climatic data and considering factors such as land management practices.^[^ ^41^ ^]^ To enhance simulation accuracy, SWAT subdivides watersheds into smaller sub‐basins, making it particularly effective for water resource management in data‐scarce regions.^[^ ^42^ ^]^ With its intuitive interface and capability to estimate water balance components, SWAT is a powerful tool for understanding and managing water resources and land use practices. In this study, the watershed was divided into sub‐watersheds and hydrologic response units (HRUs) to facilitate detailed hydrological modeling. Runoff was simulated using the soil conservation service curve number (SCS‐CN) method, while potential evapotranspiration (PET) was estimated using the Hargreaves method.^[^ ^43^ ^]^ Groundwater recharge was estimated using the SWAT model based on step‐by‐step procedures outlined in the SWAT user Manual.^[^ ^44^ ^]^ Hydrological components were estimated using the SWAT model, following the governing Equation (1).

(1) $$SW_{t}=SW_{0}+\sum_{i=1}^{t}\left(R_{\mathrm{day}}-Q_{\mathrm{surf}}-E_{a}-W_{\mathrm{seep}}-Q_{\mathrm{gw}}\right)$$

where *SW_t_* is the final soil water content (mm) at time t,*SW*_0_ is the initial soil water content at *t* = 0 (*i *= 0), *R*_day_ is the total precipitation on day *i* (mm), *Q*_surf_ is the surface runoff amount for day* i* (mm), *E_a_* is the amount of evapotranspiration on the day* i* (mm), *W*_seep_ is the inflow to the vadose zone from the soil profile on the day* i* (mm), and *Q*_gw_ is the amount of return flow on the day* i* (mm).

### 4.3 SWAT Parameterization and Setup

High‐resolution geospatial datasets, such as DEM, LULC, soil, and hydrometeorological data (climate data and steam flow) (Table 1), are required for the SWAT model to estimate hydrological components accurately. The calibration and verification of the model depend on the stream flow data. The details of geospatial and hydrometeorological datasets used in this study, along with their applications and sources, are presented in the following sections.

#### 4.3.1 Geospatial Data

The primary input for the SWAT model is the digital elevation model (DEM). The key terrain parameters such as Watershed, slope, flow length, and stream characteristics were determined from the DEM of the study area. The delineation of the area using SWAT divided the watershed into 30 sub‐watersheds (Figure 1). LULC map and its attributes are other input parameters that play a key role in influencing groundwater potential, including its availability and applicability for desired usage. In this study, LULC information was analyzed from Landsat 8 imagery using Erdas Imagine 2015 software. The ZLW is predominantly covered by eight major LULC types: cultivated land, agroforestry, shrubland, wetland, water body, plantation, afro‐alpine, and settlement. SWAT model contains default LULC codes in its databases as four‐letter digits. As a result, each LULC type was coded based on SWAT requirement (cultivated land as AGRL, settlement as URMN (Residential Med/Low Density), afro‐alpine as RNGE (Range Grasses), water body as WATR (Water Body), wetland as WETN (Wetland Non‐Forested), Agroforestry as FRST (Forest‐Mixed), Plantation as EUCA (Eucalyptus Trees) and then imported to SWAT project for HRUs definition and watershed characterization.

Soil map and soil attribute table are the critical inputs for SWAT in simulating water balance components, defining HRUs, and watershed characterization. In this study, the physical and chemical properties of the region's major soil types were used as one input for the model. Eight major soil types in the ZLW, such as Leptosols, Vitric Andosols, Calcaric Fluvisols, Eutric Cambisols, Rhodic Nitisol, Eutric Vertisol, Haplic Luvisols are common in the region.^[^ ^32^ ^]^ Information on soil texture, hydraulic conductivity, available water capacity, bulk density, and water movement are essential in simulating infiltration and recharge. The DEM, slope, LULC, and soil data were imported, overlaid, and integrated into the SWAT database for sub‐watershed characterization and HRUs definitions. To ensure an accurate representation of spatial heterogeneity, the threshed values LULC (20%), soil (10%), and slope (20%) were assigned in the model. These thresholds were essential to refine the spatial variability of watershed characteristics for improved hydrological modeling.^[^ ^45^ ^]^

#### 4.3.2 Hydro‐Climate Data

Hydro‐climate data (HC) are crucial for simulating water balance using the SWAT model. The long‐term daily weather data, including temperature, precipitation, wind speed, and humidity are essential climate data for the SWAT model. In this study, the conventional climate data was supplemented with freely available online climate data. The daily maximum and minimum temperatures, and precipitation records from 14 weather stations (1982–2016) were used. The missing values were addressed using the arithmetic mean and normal ratio (NR) techniques to ensure data reliability. Stream flow records from Katar and Meki river gauging stations (MoWE, are pivotal observed hydrological data employed to calibrate and validate the model. The data were thoroughly checked for sufficiency, homogeneity, and completeness. Finally, the observed and simulated streamflow were compared, ensuring the model accurately captured the water balance component in the watershed.

### 4.4 Sensitivity Analysis

Sensitivity analysis is a critical step in hydrological modeling and its pivotal to identify the parameters that most influence model outputs. In this study, sensitivity analysis was conducted using the Calibration and Uncertainty Program (SWAT‐CUP), specifically employing the Sequential Uncertainty Fitting Version 2 (Sufi – 2) algorithms.^[^ ^46^ ^]^ Daily streamflow data from the Katar and Meki rivers (1982–2016) were used for sensitivity analysis. The global sensitivity analysis (GSA) techniques of SWAT‐CUP were used to determine the key parameters affecting model output, thereby improving calibration accuracy. GSA is particularly effective for complex models, as it accounts for parameter interactions and uncertainty.^[^ ^46^ ^]^ Relative to local sensitivity analysis, GSA provides a more comprehensive and robust evaluation of parameter influence.^[^ ^44^, ^47^ ^]^ The parameters were then ranked based on their t‐statistic values, with higher values indicating greater sensitivity. In the sensitivity ranking, rank 1 indicates the most influential parameter owing to small changes in its value significantly impact on streamflow simulation. Based on this analysis Curve Number (CN2) is the most sensitive parameter and Ranked 1, whereas channel hydraulic conductivity (CH_K2) has a minimal effect (Rank 14) on stream flow in the Watershed (**Table** **2**).

### 4.5 Calibration, Validation and Performance Evaluation

**Figure 3:**
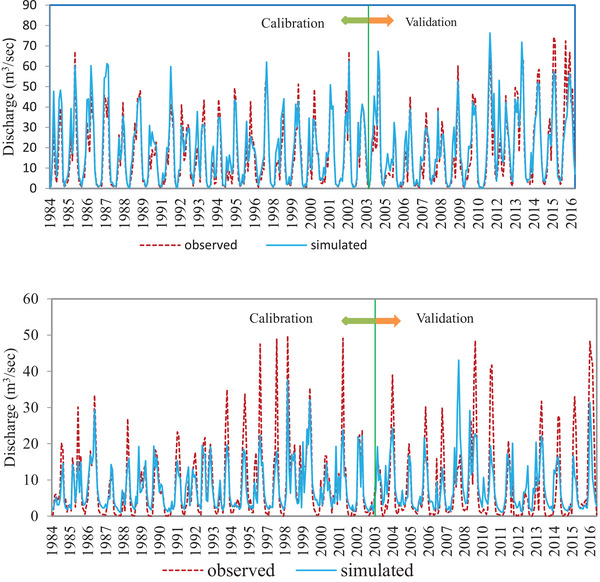
Monthly observed and simulated streamflow during the calibration (1984–2003) and validation periods (2004–2016) for: a) Katar River, b) Meki River, and c) Katar River, d) Meki River.

Calibration is the process of adjusting some of the model's input parameters to achieve a better agreement between the observed and simulated stream flow. It is a critical step to ensure that the model accurately reflects actual conditions, enhancing its ability to capture reliable predictions for decision‐making and predictions. The calibration and validation are carried out employing the Calibration and Uncertainty Program (SWAT‐CUP) based on Sequential Uncertainty Fitting version 2 (Sufi – 2).^[^ ^48^ ^]^ Daily streamflow records (1984 to 2003) and (2004 to 2016) were used for calibration and validation of the SWAT model respectively based on manual and automatic techniques. The first two years (1982–1983) were used as a warm‐up period to improve water balance simulation.^[^ ^46^ ^]^ Two calibration sites downstream were chosen to capture key water processes in the watershed. The validation was using the same data stream without modifying the calibration parameters, based on scientific recommendation (**Table** **3**). The observed versus simulated hydrograph (**Figure** **3**) shows the best match in rising and recession limbs, revealing the applied SWAT model captured seasonal variation well. Furthermore, three performance indicators: coefficient of determination (R^2^), Nash–Sutcliffe Efficiency (NSE) Equation (3), and percent bias (PBIAS) were used to evaluate the performance of the model. These performance measures are internationally used due to their universality and dependability.

**Table 3:** Performance evaluation criteria for the selected parameters. (Moraise et al., 2015).

Rating	*R* ^2^	NSE	PBIAS [%]
Very good	0.75–1	0.75–1	< ±10
Good	0.65–0.75	0.65–0.75	±10 –±15
Satisfactory	0.5–0.65	0.5–0.6	±15–±25
Not satisfactory	< 0.5	≤ 0.5	>±25

An R^2^ value relates the observed stream values to the simulated stream flow values at calibration and validation sites and the NSE value assesses the magnitude of the variance of the residuals,^[^ ^49^ ^]^ against the measured steam flow data. *R* ^2^, which measures the amount of variation explained by the model, provides a metric for how good the fit of the model's simulated values is to the observed values, with a value between 0 and 1 Equation (2).

(2) $$R^{2}=\frac{{\left[\sum_{i}^{n}\left(Q_{obs}-Q_{mo}\right)\left(Q_{s}-Q_{ms}\right)\right]}^{2}}{\sum_{i}^{n}{\left(Q_{obs}-Q_{mo}\right)}^{2}\sum_{i}^{n}{\left(Qs-Qms\right)}^{2}\;}$$

where *Q_obs_* is the observed flow (m^3^ s^−1^) and *Q_mo_* Is the mean of the observations, *Q_s_* is the simulated flow (m^3^ s^−1^) and *Q_ms_* is the mean of simulation. N stands for the total number of observations.

(3) $$NSE=\frac{\sum_{i=1}^{n}\left(Q_{obs}-Q_{mo}\right)2}{\sum_{i=1}^{n}\left(Q_{obs}-Q_{ms}\right)2}$$

NSE values range from 0 to 1. Anything greater than 0 demonstrates good performance of the model, while anything less than 0 is a very low fit, and PBIAS is the percent bias Equation (4). PBIAS = 0 indicates no bias and values between −25 and 25 reflect very good model performance.

(4) $$PBIAS=100\times \frac{\sum_{i=1}^{n}\left(Q_{ms}-Q_{s}\right)i}{\sum_{i=1}^{n}Q_{ms},i}$$

### 4.6 Multi‐Criteria Decision Analysis (MCDA)

A GIS‐based MCDA technique was applied to determine the groundwater potential zones in the ZLW. It is an effective tool for water resource assessment, prioritization, and management.^[^ ^18^, ^50^, ^51^, ^52^, ^53^ ^]^ Among the different MCDA techniques, the analytical hierarchical process (AHP) was selected due to its wide application in Ethiopia,^[^ ^4^, ^23^, ^40^, ^54^, ^55^ ^]^ and globally.^[^ ^12^, ^56^, ^57^, ^58^ ^]^ Key factors controlling groundwater movement, occurrence, and storage were identified for the lake basin: Recharge, elevation, lithology, lineament density, and drainage density (**Table** 4).

**Table 4:** AHP rating scale.

Rating	Level of significance	Explanations
1	Equal Importance	Both elements hold the same level of significance.
3	Minor Difference	One factor is slightly more significant than the other.
5	Major Difference	One factor is considerably more significant than the other.
7	Clear Dominance	One factor is distinctly more important than the other.
9	Total Dominance	One element is overwhelmingly more important than the other.
2, 4, 6, 8	Intermediate Ratings	Values that represent a range between two adjacent judgments.

### 4.7 Thematic Layers Preparation

Groundwater potential mapping was conducted using GIS and AHP with 12.5 m spatial resolution, incorporating Recharge, lithology, elevation, lineament density, and drainage density. Environmental factors were rated and weighted based on their impact on recharge.^[^ ^59^, ^60^ ^]^ The groundwater potential map was created using weighted linear combination (WLC) techniques and validated using springs, wells, and borehole sites. The results were further improved using the receiver operating characteristic (ROC) curve and area under the curve (AUC) analysis. A detailed analysis of environmental factors is provided in the following sections.

#### 4.7.1 Recharge

**Figure 4:**
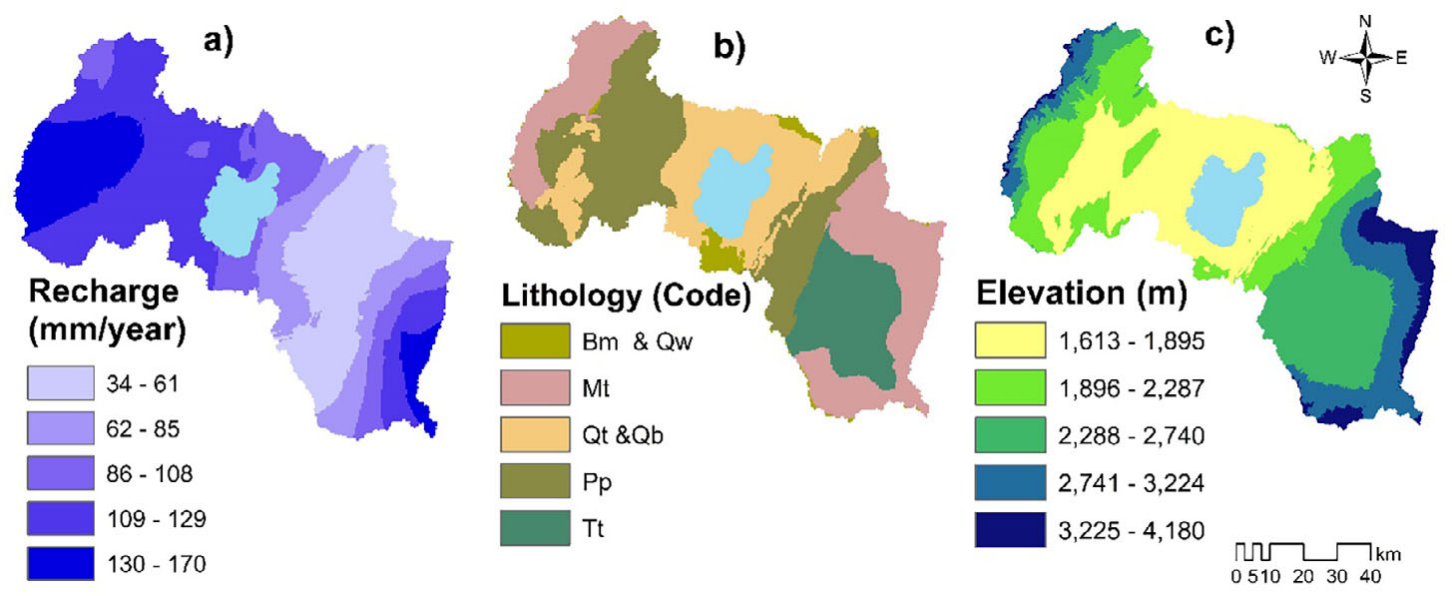
Maps of thematic factors: a) Recharge, b) Lithology, c) Elevation. Where Qw: Basaltic lava flow and scoria cones, Bm; Gneiss and sandstone, shale, marl, Mt; Chilalo Shield volcanoes: Basalt, Trachytes, mugearite, and phonolite, Tt: Volcanite of plateaux; rhyolitic ignimbrite and tuff Pp: Rift floor Ignimbrite; peralkaline rhyolitic ignimbrite, very lowly welded ignimbrite, tuff, ash, minor Basalt, Qt: Lacustrine sediments: Sand, silt, pyroclastic deposit, and Diatomite, Qb: Rhyolite lava, pumice, pantelleritic obsidian flows.

Recharge was determined as a thematic factor (Figure 3a) to assess groundwater potential conditions, serving as a key factor in understanding the region's water resources. Using the SWAT model, recharge was estimated and subsequently reclassified in GIS based on its relevance to groundwater potential. **Figure** **4**a shows the spatial distribution of mean annual groundwater recharge across the ZLW, classified into five categories: Very low recharge (34–61 mm), low recharge (62–85), moderate recharge (86–108 mm), high recharge (109–129) and very high recharge (130–170 mm). High recharge areas were assigned higher groundwater potential values, while low recharge areas received lower values (**Table** **5**).

**Table 5:** Pairwise comparison matrix (PCM).

Factor	Recharge	Lithology	Elevation	Lineament density	Drainage density	Wt. [%]
Recharge	1	2	3	7	9	45
Lithology	1/2	1	2	5	7	28
Elevation	1/3	1/2	1	3	5	17
Lineament density	1/7	1/5	1/3	1	2	6
Drainage density	1/9	1/7	1/5	1/2	1	4
Column sum	2.087	3.843	6.533	16.5	24	100

#### 4.7.2 Lithology

Existing geological maps of the study area were utilized to delineate and characterize groundwater recharge potential within the watershed. The lithological characteristics have a direct impact on groundwater recharge.^[^ ^61^, ^62^ ^]^ In the ZLW, groundwater potential was assessed based on permeability and recharge capacity. *Q*_w_ (basaltic lava flows and scoria cones) has very low potential 1) due to very low permeability. Mt (Chilalo Shield volcanoes) is rated low 2) for low permeability. Tt (rhyolitic ignimbrite and tuff) has moderate potential (3). Pp (rift floor ignimbrite) is rated high 4) due to high permeability. Qt (lacustrine sediments and rhyolite lava/pumice/obsidian flows) have very high potential (5), playing a crucial role in groundwater recharge with excellent permeability (Figure 4b).

#### 4.7.3 Elevation

Elevation map (EL) is analyzed from DEM (https://search.asf.alaska.edu) of the study area using ArcGIS 10.8. EL is the manifestation of topographic gradients that influence surface runoff and groundwater recharge potential. EL layer was classified into five subcategories based on its impact on groundwater potentiality. The areas of the watershed with higher elevations are characterized by steep slope angles and with poor groundwater potential (raked 1), while flatter areas around the center/rift floor lands of the watershed (Figure 4c) are associated with very high pondwater potential (ranked 5) (Table 7).

#### 4.7.4 Lineament Density

**Figure 5:**
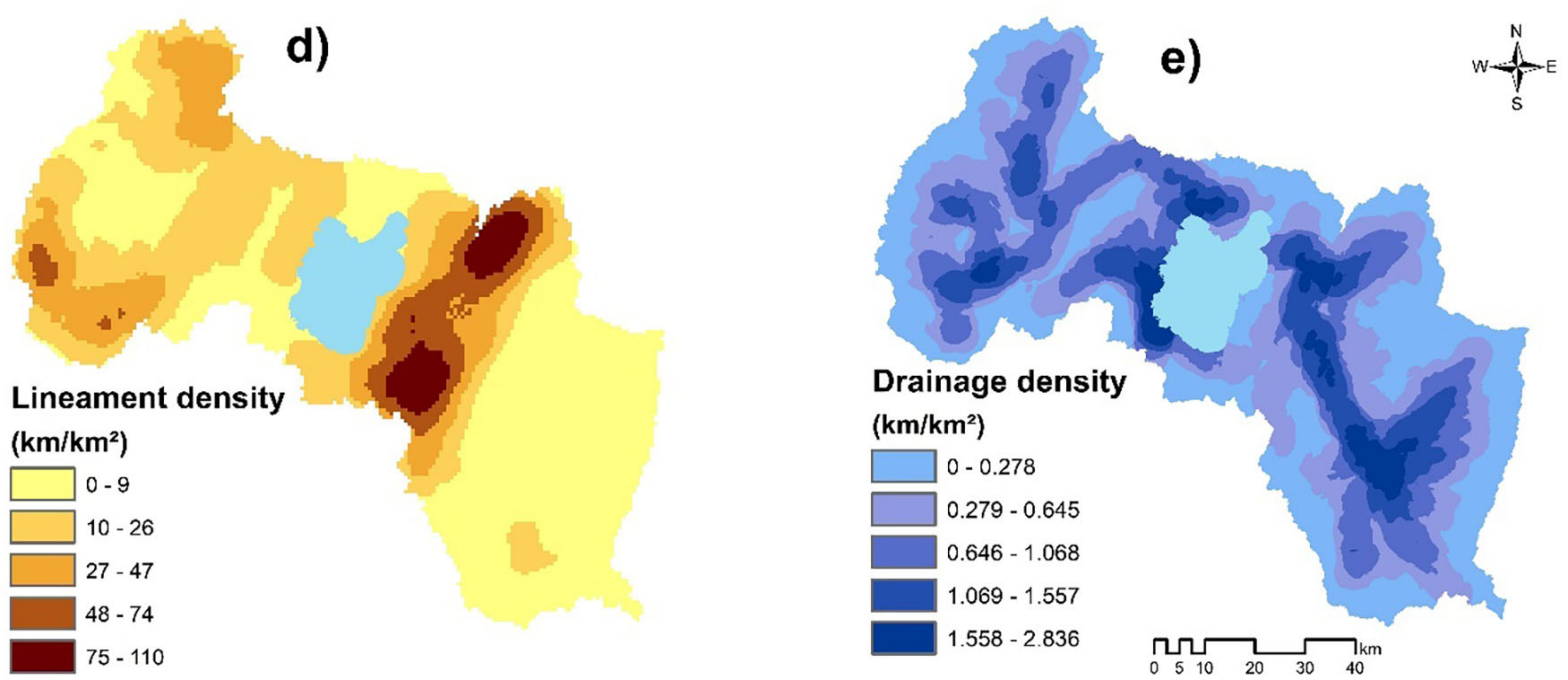
Maps of thematic factors: a) Lineament density, and b) Drainage density.

Lineament density (LD) is crucial for groundwater potential in the ZLW. In this study, LD was analyzed with Geomatic 2018 and ArcGIS 10.8 from Landsat 8 imagery (2023) and geological maps. Five lineament classes were rated with different groundwater potential indexes (1 to 5) based on their conditions to groundwater potential (**Figure** **5**d). These features are pivotal for groundwater flow and storage.^[^ ^23^ ^]^ LD indicates the connectivity and density of fractures/lineaments. It's a ratio of the total length of the lineament in the watershed to the area of the watershed Equation (5):

(5) $$LD=\frac{\sum_{i=1}^{n}L_{i}}{A}$$

where *Li* *d*Otes the length of the lineament and *A* represents the Watersheds area.

The cracks and fissures facilitate water infiltration, enhancing groundwater recharge. The high LD is linked to very high groundwater potential as it is directly correlated with the groundwater potentiality.^[^ ^62^ ^]^ The rift floor land and areas surrounding the ZL have higher LD and higher groundwater potential (ranked 5).

#### 4.7.5 Drainage Density

Drainage density (DD) was identified as a key factor affecting groundwater in the ZLW. It was derived from the DEM and indicates how runoff impacts groundwater potentiality. Higher DD favors runoff, while lower DD is linked to better recharge potential.^[^ ^62^ ^]^ DD was calculated using GIS tools based on Equation (6) and classified into five classes based on its relation with groundwater potentiality.

(6) $$DD=\frac{\sum TLS}{TAW}$$

The regions of the study area with higher DD were assigned lower groundwater prospect rates (1), while areas with DD were given higher scores (5) (Figure 5e). This method effectively captures the relationship between surface runoff and groundwater replenishment across the watershed.

### 4.8 Weight Assignment and Normalization

To determine the relative weight of the thematic layers, a series of logical steps were performed, starting with the Saaty scaling,^[^ ^59^ ^]^ of the selected thematic layers with their sub‐classes, pairwise comparison matrix (PCM), weights normalization (**Table** **6**), and finally consistency ratio computation was computed. Saaty's 1–9 scaling was used to assign weights to thematic layers based on their impact on groundwater potential. A weight of 9 indicates a high impact, while 1 indicates a low impact. The AHP and a PCM were applied to define groundwater recharge potential. This technique has been applied by many authors for groundwater potential investigations.^[^ ^6^, ^18^, ^54^, ^63^ ^]^

**Table 6:** Normalized pairwise Comparison matrix (NPCM).

Factor	Recharge	Lithology	Elevation	Lineament density	Drainage density	Normalized weight
Recharge	0.479	0.52	0.459	0.424	0.375	0.45
Lithology	0.24	0.26	0.306	0.303	0.292	0.28
Elevation	0.16	0.13	0.153	0.182	0.208	0.17
Lineament density	0.069	0.052	0.051	0.061	0.083	0.06
Drainage density	0.053	0.037	0.031	0.03	0.042	0.04
Criteria evaluation	*λ*_max _= 5.05, CI = 0.012, CR = 0.011 < 0.1: Acceptable					

### 4.9 Consistency Evaluation

The AHP consistency ratio (CR) check's reliability, with values < 0.1 considered valid. In this study, the CR was 0.011, well below the 0.1 threshold (**Tables** 7 and 8).^[^ ^64^ ^]^

**Table 7:** Groundwater potential factors, their classes, rating values, and rank.

Thematic layers	Rank	Features/classes	Normalization	Groundwater prospect	Weight [%]
Recharge (mm year^−1^)	1	34–61	0.022	Very low	45
	2	62–85	0.044	Low	
	3	86–108	0.066	Moderate	
	4	109–129	0.089	High	
	5	130–170	0.111	Very high	
Lithology	1	Gneiss and sandstone, shale, marl	0.036	Very low	28
		Basaltic lava flow and scoria cone			
	2	Chilalo Shield volcanoes: Basalt, trachyte, and mugearite	0.071	Low	
	3	Volcanite of plateaux; rhyolitic ignimbrite and tuff	0.107	Moderate	
	4	Rift floor Ignimbrite; peralkaline rhyolitic ignimbrite	0.143	High	
		welded ignimbrite, tuff, ash, minor basalt			
	5	Rhyolite lava, pumice, pantelleritic obsidian flows	0.178	Very high	
		Lacustrine sediments: sand, silt, pyroclastic deposit			
Elevation	1	3225–4180	0.060	Very low	17
	2	2741–3224	0.120	Low	
	3	2288–2740	0.180	Moderate	
	4	1896–2287	0.240	High	
	5	1613–1895	0.300	Very high	
Lineament density	1	0–9	0.061	Very low	6
	2	10–26	0.121	Low	
	3	27–47	0.182	Moderate	
	4	48–74	0.242	High	
	5	75–110	0.303	Very high	
Drainage density	1	1.558–2.836	0.011	Very low	4
	2	1.069–1.557	0.022	Low	
	3	0.646–1.068	0.032	Moderate	
	4	0.279–0.645	0.043	High	
	5	0–0.278	0.054	Very high	

**Table 8:** Saaty's consistency indices.

Number of thematic factors [*n*]	1	2	3	4	5	6	7	8	9	10
Random index value [*RI*]	0	0	0.58	0.9	1.12	1.24	1.32	1.41	1.45	1.49

The consistency index (CI) was computed using Equation (7).

(7) $$CI=\frac{\lambda \max-n}{n-1}$$

The CI was 0.011, with *λ*_max_ = 5.05, confirming the reliability of the PCM. (Khan et al., 2023)

(8) $$CR=\frac{CI}{RI}$$

The CR value of 0.011 is well below the 0.1 threshold, showing that the comparisons are consistent and reliable.

### 4.10 Identification and Validation of Groundwater Potential Zone Map

The GIS‐based weighted overlay analysis (WOA) was used using ArcGIS 10.8 to delineate GWPZs. WOA integrates multiple thematic layers with assigned weights, making it an effective spatial analysis tool for groundwater potential mapping and site suitability assessment^[^ ^5^, ^12^ ^]^ WOA method assigns a 1–5 scale to sub‐parameters, reclassified raster maps, and computes weighted values of each raster cell^[^ ^64^ ^]^ The groundwater potential zone index (GWPZI) is then computed using the WLC technique Equation (7), a widely applied technique in groundwater studies.^[^ ^64^, ^65^, ^66^ ^]^

(9) $$GwpzI=\sum_{1}^{n}W_{j}\times R_{i}$$

where *W*_j_ – normalized weight for each thematic factor, *R*_i_ – rank of sub‐thematic factors.

The final GWPZ raster was categorized into five groundwater potential classes using natural break classification techniques within a GIS environment.

The observed data such as safe yield, boreholes, and spring locations can be used to validate groundwater potential maps.^[^ ^64^, ^66^, ^67^ ^]^ In this study, the observed hydrological data from 60 springs and well sites were used to validate the groundwater potential map. This water point data was converted into a shapefile using ArcGIS 10.8. These points were overlaid on the GWPZ raster layer in the ArcMap view. Validation was then carried out by using the ROC tool in combination with the GIS GWPZ map. These methods are commonly used for groundwater potential validation by many researchers.^[^ ^20^, ^51^ ^]^

### 4.11 Integration of Groundwater Potential with Drinking Water Quality Index

Groundwater can be considered a potential resource only if its quality is appropriate for the intended use. Along with assessing groundwater potential, it is also necessary to assess groundwater quality before it is used for domestic, irrigation, or industrial purposes (Suresh et al., 1991). Water quality indexes (WQIs), based on the chemical composition of the water, are efficient techniques for determining overall water quality by aggregating several water quality parameters into a single number that assists managers and decision‐makers.^[^ ^50^, ^68^ ^]^ Evaluated the suitability of groundwater in the Ziway Lake Watershed for human consumption by considering physical, major ions, and trace elements. This study documented that groundwater water quality is highly variable across the watershed, and groundwater is generally of excellent quality for drinking use in the headwater regions of the watershed and gradually becomes extremely unsuitable toward the rift floor. The extremely unsuitable water quality in the lake region is mainly associated with the co‐occurrence of multiple toxic elements in groundwater from the Quaternary sediments and rhyolitic volcanic aquifers.

To identify potential zones for successful groundwater development in the area, the groundwater quality map developed using the WQI approach is integrated with the groundwater potential map established using a GIS‐based MCDA technique. The integration of spatial data layers was achieved through the application of the Intersect function within the spatial analysis module of the GIS package. Finally, by integrating the groundwater potential zone classes with the groundwater quality classes, areas of both groundwater potential and groundwater quality and suitability were delineated.

## 5 Result and Discussion

### 5.1 Groundwater Recharge

Groundwater recharge (Re) in the ZLW was estimated using the SWAT model. The analysis results of the model indicate ranges of mean annual recharge rates: very low (34–61 mm year^−1^), low (62–85 mm year^−1^), moderate (86–108 mm year^−1^), high (109–129 mm year^−1^), and very high (130–170 mm year^−1^). The midpoints of these ranges are 47.5, 73.5, 97, 119, and 150 mm year^−1^. The mean recharge rate is ≈97.4 mm year^−1^, with a standard deviation of 34.04 mm year^−1^, revealing that recharge rate variability across the region. Areas with higher recharge rates are associated with higher rainfall and permeable soil types, and high facilitated infiltration. In contrast, lower recharge zones are liked with regions of higher evaporation rates, lower precipitation, and less permeable soils.^[^ ^32^ ^]^ These findings highlight the importance of strategic groundwater management, focusing on areas with higher recharge potential, to ensure sustainable water resources for the future.^[^ ^42^, ^45^ ^]^ The SWAT model demonstrated strong performance in estimating recharge (**Table** **9**). The performance evaluation results reveal at Katar River, R^2^ values are 0.86 during calibration and 0.84 during validation phases with NSE values of 0.84 and 0.83 respectively.

**Table 9:** Goodness fit is considered for the model.

Gauging sites	Objective functions			
	Model phase	*R* ^2^	NSE	PBIAS
Katar	Calibration	0.86	0.84	−13.7
	Validation	0.84	0.83	3.2
Meki	Calibration	0.64	0.62	−12.0
	Validation	0.79	0.78	5.9

PBIAS was −13.7 during calibration with slight underestimation and 3.2 during validation with slight overestimated results. At Meki river *R* ^2^ is 0.64 during calibration 0.79 during validation, with NSE 0.62 and 0.78, and PBIAS of ≈−12 and 5.9 (Table 9).

### 5.2 Groundwater Potential Zone

**Figure 6:**
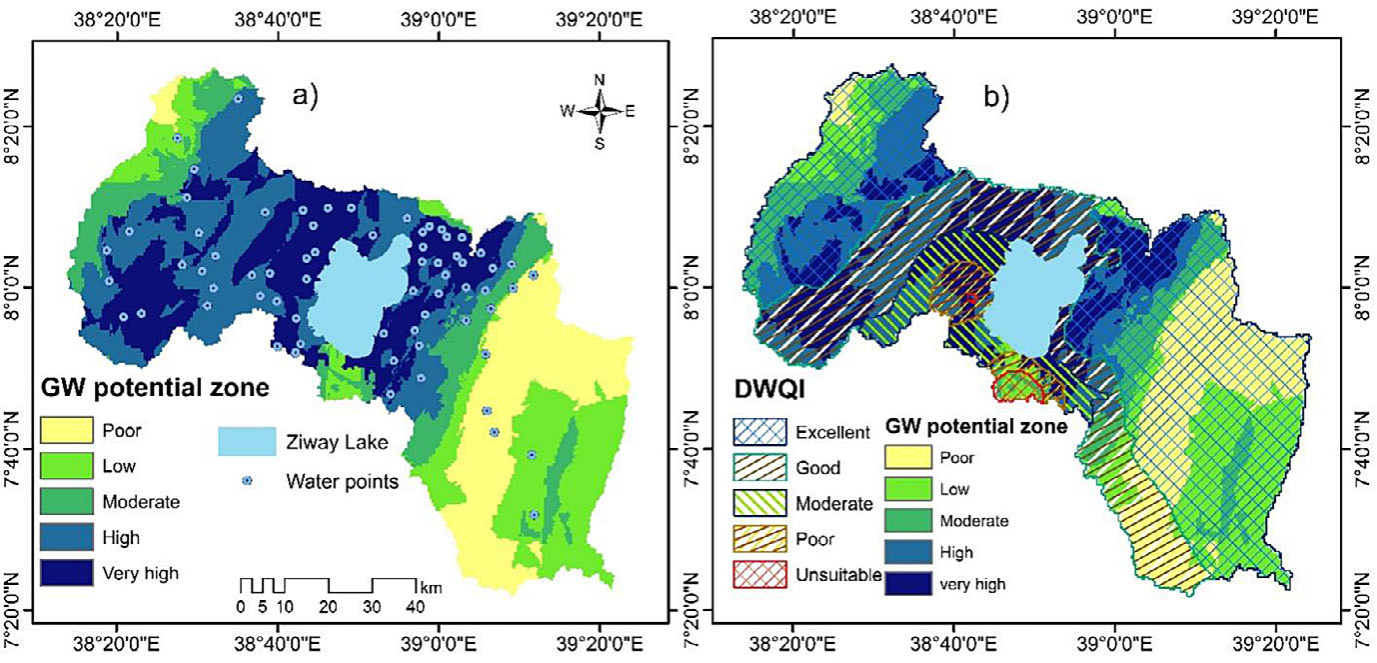
Groundwater potential zone map (a) and an overlain groundwater potential zone map with drinking groundwater quality indices map (b).

The groundwater potential across the Ziway Lake Watershed (ZLW) varies significantly, as illustrated in **Figure** **6**. The region is classified into five GWPZs: Very low (1277 km^2^, 18.01%), low (1249 km^2^, 17.62%), moderate (854 km^2^, 12.05%), high (2009 km^2^, 28.34%), and very high (1700 km^2^, 23.98%). Groundwater potential across the region is strongly influenced by recharge (Re), lithology (Lith), elevation (EL), lineament density (LD), and drainage density (DD). The GWPZ map (Figure 6) shows that much of the western and central parts of the watershed, particularly low‐lying rift floor areas, have high to very high groundwater potential. These areas are linked with recent basalts and fractured ignimbrites.^[^ ^39^ ^]^ Very high groundwater potential zones (23.98 (% of the total area) correspond to areas with optimal conditions for groundwater accumulation, including highly permeable lithologies, low elevation, and very high lineament densities (LD). Geological features such as faults and volcanic formations, analyzed through lineament density (LD), play a critical role in controlling groundwater movement, storage, and availability.^[^ ^36^ ^]^

These factors highlight how groundwater moves and accumulates efficiently in certain regions. High potential areas (28.34%) are characterized by favorable conditions, including high recharge rates, permeable rocks, low to moderate elevation, and fractures that promote groundwater storage and movement. These areas have high permeability, driven by joints, faults, vesicles, and scoria fragment size, with rates between 10 and 20 m day^−1^. Additionally, very high to high GWPZs are associated with lacustrine sediments in low‐slope areas, intensely fractured. Despite the low recharge, these aquifers can store and transmit substantial amounts of water. In contrast, moderate potential regions (12.05%) are located between the high and low groundwater potential zones.

These areas cover a limited portion of the region and are associated with low LD, high DD, and steep slopes in rift escarpment areas, though their efficiency is lower relative to high and very high potential zones. Low potential zones (17.62%) (depicted in light green in Figure 6) are mainly found along the margins of the study watershed. These areas exhibit moderate recharge, less permeable rocks, and higher elevations, all of which hinder water retention and movement. Finally, very low potential zones (18.01%) demonstrate the least favorable conditions for groundwater, with low recharge, impermeable lithologies, high elevation, and low LD. These factors restrict groundwater storage and movement.

The spatial distribution of GWPZ in the ZLW is closely linked to lithological characteristics and physiographic features. Specifically, areas with permeable lithologic units, low topography (such as rift floor land (LFL)), and high lineament density exhibit the highest groundwater recharge potential (Figure 6). Areas with basaltic lava flows and scoria cones (Qw), like those around the Chilalo Shield volcanoes (Mt) are linked with low groundwater potential. This result demonstrates that Qw and Mt lithologic units have very low to low permeability and facilitate surface runoff than infiltrations. This zone is mainly found around the highlands and margins of the watershed. In contrast, the central area, near the ZL is covered with rhyolitic ignimbrite, tuff (Tt), rift floor ignimbrite (Pp), lacustrine sediments, and rhyolite lava/pumice/obsidian flows (Qt), which have moderate groundwater potential. This indicates that these rocks are pervious enhancing groundwater potential/infiltration. In this region, GWPZs are classified as high to very high, owing to great potential for replenishing aquifers. The areas with very high fracture density (LD) show better groundwater movement and availability. However, steep slopes, high elevations, and high drainage density are mostly found along the escarpment and highlands of the watershed (Figure 6a). These factors are linked to lower recharge potential (Re), as they increase surface runoff and reduce water infiltration, especially in areas with less permeable soils and very low fracture density.

This study outlines a sustainable water management framework for the ZLW by identifying critical groundwater recharge zones. It combines techniques like artificial recharge, land‐use planning, and AHP to assess groundwater potential. About 52.32% of the area has a high groundwater potential, mainly in lower elevations with high recharge rates, while 47.68% shows lower potential due to very low recharge and impermeable rocks. The findings emphasize boosting recharge/implementing artificial recharge mechanisms in lower potential zones and protecting high‐potential areas for effective groundwater management and policy development.

### 5.3 Validation of Groundwater Potential Map

**Figure 7:**
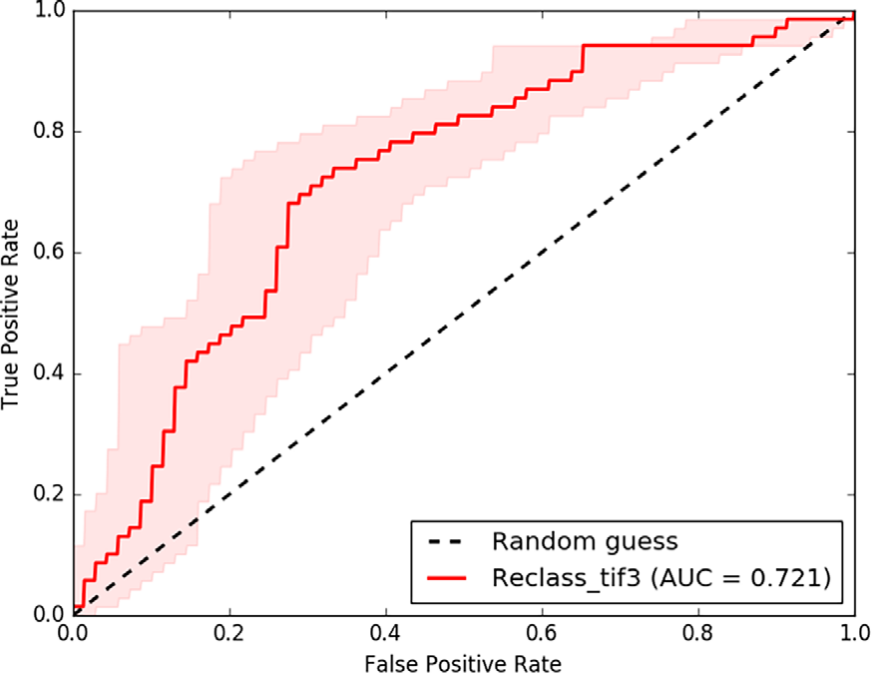
ROC plot for validation of the Groundwater potential map.

The accuracy of derived GWPZs was validated using receiver operating characteristics (ROC) curve analysis, achieving an area AUC of 72.1% (**Figure** **7**). This confirms that the groundwater potential map reliably represents real‐world conditions. A similar level of accuracy has been reported in previous studies at various watershed scales.^[^ ^54^, ^69^ ^]^ The high AUC value demonstrates the effectiveness of the AHP in mapping groundwater potential zones. The study underscores the importance of integrating geospatial data, hydrogeological parameters, and machine learning approaches to further refine groundwater potential assessments.

### 5.4 Integrated Groundwater Potential zone and Drinking Water Quality mapping

The analysis of GWPZ in conjunction with the drinking water quality indices (DWQI) highlights areas with higher groundwater recharge potential associated with good water quality. The resultant groundwater potentiality map indicated that very high GWPZ maps cover ≈652.72 km^2^ (9.21%) of excellent DWQI and 688.95 km^2^ (9.72%) of good suitability for drinking. Likewise, the high GWPZ occupies ≈707.1 km^2^ (9.97%) of excellent quality and 771.5 km^2^ (10.88%) of good groundwater quality for drinking (**Table** **10**). These findings emphasize the importance of protecting the GWPZ zone to ensure a sustainable and high‐quality water supply.

**Table 10:** Distribution of areas across different groundwater potential zones.

Groundwater potential zones	Weight class	Area [km^2^]	Area [%]
Very low groundwater potential zone	1	1277	18.01
Low groundwater potential zone	2	1249	17.62
Moderate groundwater potential zone	3	854	12.05
High groundwater potential zone	4	2009	28.34
Very high groundwater potential zone	5	1700	23.98

The results (Table 10) reveal a strong correlation between high groundwater potential and good water quality, identifying these regions as key priorities for sustainable water management. While areas with low GWPZ exhibit lower groundwater potential, they still make a significant contribution to groundwater quality, with 1060.83 km^2^ (14.96%) classified as having excellent‐quality groundwater. However, the analysis reveals localized challenges. Notably, 72.34 km^2^ (1.02%) of high groundwater GWPZ areas contain very low‐quality groundwater, while 1.82 km^2^ (0.03%) of very high GWPZ regions are classified as unsuitable for consumption. The combination of the GWPZ zone map with the DWQI map reveals that 2808.03 km^2^ (39.72%) of the study area falls under very high and high GWPZs with good to excellent DWQI. However, ≈234.74 km^2^ (3.30%) of these zones exhibit very low or unsuitable water quality. In contrast, areas classified as moderate and low GWPZs, covering 1381.46 km^2^ (19.39%), are predominantly characterized by excellent drinking water quality (**Table** 1, 11).

**Table 11:** Integration of groundwater potential zones and drinking water quality indices.

An integrated groundwater potential zone and drinking water quality index	Area [km^2^]	Area [%]
very high groundwater potential with excellent quality	652.72	9.21
very high groundwater potential with good quality	688.95	9.72
very high groundwater potential with moderate quality	330.03	4.66
very high groundwater potential with very low quality	134.71	1.90
very high groundwater potential with unsuitable quality	1.82	0.03
high groundwater potential with excellent quality	707.10	9.97
high groundwater potential with good quality	771.50	10.88
high groundwater potential with moderate quality	354.20	5.00
high groundwater potential with very low quality	72.34	1.02
high groundwater potential with unsuitable quality	1.15	0.02
moderate groundwater potential with excellent quality	710.50	10.02
moderate groundwater potential with good quality	91.50	1.29
moderate groundwater potential with moderate quality	4.91	0.07
moderate groundwater potential with very low quality	23.26	0.33
moderate groundwater potential with unsuitable quality	22.57	0.32
very low groundwater potential with excellent quality	988.20	13.94
very low groundwater potential with good quality	287.13	4.05
low groundwater potential with excellent quality	1060.83	14.96
low groundwater potential with good quality	126.19	1.78
low groundwater potential with moderate quality	4.78	0.07
low groundwater potential with very low quality	20.86	0.29
low groundwater potential with unsuitable quality	33.84	0.48

This study emphasizes the need for strategic groundwater management, particularly in areas with high groundwater potential but very low water quality. It identifies key conservation zones based on the alignment of recharge potential and water quality. A holistic approach is recommended to address local water quality challenges, incorporate all recharge zones, and safeguard high‐potential areas for long‐term water security. Despite its valuable insights, this study has certain limitations. These include potential bias from the AHP method, uneven well distribution in the GWPZ map, and the reliance on secondary climate and streamflow data. Future research could enhance accuracy by incorporating geophysical methods for more precise groundwater assessments, machine learning (ML) algorithms to improve predictive modeling, and google earth engine for large‐scale spatial analysis. Additionally, analyzing temporal trends in groundwater potential zones would offer valuable insights into long‐term groundwater resource dynamics, particularly in response to anthropogenic activities and climate change.

## 6 Conclusion

This study successfully created a GWPZ map in the ZLW using SWAT, RS, GIS, and MCDA. The groundwater recharge was estimated using the SWAT model, and the map was validated through ROC analysis. Integration with the Drinking Water Quality Index provided a comprehensive assessment of both groundwater potential and water quality. Over 50% of the watershed falls within moderate to very high groundwater potential zones crucial for developing and adopting practical groundwater management strategies. Groundwater potential is classified as very low (18.05%), low (23.6%), moderate (21.89%), high (19.64%), and very high (16.81%). The GWPZ map combined with the DWQI reveals that 39.72% of the area (2808.03 km^2^) has high to very high potential with good to excellent water quality. Moderate and low potential zones (19.39%, 1381.46 km^2^) generally show excellent water quality, underscoring the need for targeted management in areas with very low water quality. This study emphasizes the importance of environmental factors in groundwater assessments. Using AHP, it identified key recharge areas to guide effective water management and conservation. The combined applications of SWAT and GIS‐based MCDA models offer a practical framework for sustainable groundwater development and management. These findings provide a valuable tool for integrated water resource management in the Ziway Lake watershed and can be applied globally to regions with similar conditions for effective conservation and development.

## Conflict of Interest

The authors declare no conflict of interest.

## Data Availability

The data that support the findings of this study are available from the corresponding author upon reasonable request.
